# Reduced pupillary reward sensitivity in Parkinson’s disease

**DOI:** 10.1038/npjparkd.2015.26

**Published:** 2015-12-17

**Authors:** S G Manohar, M Husain

**Affiliations:** 1 Nuffield Department of Clinical Neurosciences, University of Oxford, Oxford, UK

## Abstract

Abnormalities in reward processing may be a critical part of understanding nonmotor manifestations of Parkinson’s disease (PD). Dysfunction in dopaminergic pathways, which signal upcoming rewards, might result in altered motivation by incentives. To examine this proposal, we studied 16 patients with PD, both ON and OFF their normal dopaminergic medication, comparing them with healthy controls. Participants performed a speeded saccade task to obtain monetary rewards. Crucially, we manipulated the reward available from trial to trial, by presenting an auditory incentive precue before each saccade. The effects of incentives on pupil dilatation (an index of autonomic response) were measured. Individuals with PD showed diminished autonomic reward effects, compared with age-matched controls. When tested ON medication, pupil responses to reward increased, demonstrating that dopaminergic drugs can restore reward sensitivity. These findings reveal blunted autonomic responses to incentives in PD, which can be modulated by dopaminergic drugs.

## Introduction

The processing of reward information, and in particular reward sensitivity, has become central to understanding several aspects of Parkinson’s disease (PD) and the role of dopamine.^1,2^ Learning from reward is altered in PD in a dopamine-dependent manner,^3^ with weaker reinforcement in reversal learning.^4^ These effects can be explained in terms of unexpected rewards, signaled by dopamine, driving striatal plasticity.

Abnormal responses to reward have been implicated in both impulse control disorders and apathy.^5–7^ It is likely that these nonmotor features have a dopaminergic basis.^8^ The direct effect of rewards on action may be hard to observe, however, as dopamine depletion itself results in motor dysfunction.

Pupillometry presents a unique opportunity to study reward effects independently of motor responses, providing a “window to the preconscious”.^9^ In healthy people, reward and motivation by rewards lead to pupillary dilatation, presumably by effects on arousal and attention.^10^ Reward, signaled by dopamine, may dilate the pupil by increasing sympathetic outflow at the locus coeruleus energizing upcoming actions.^11^

**Figure 1:**
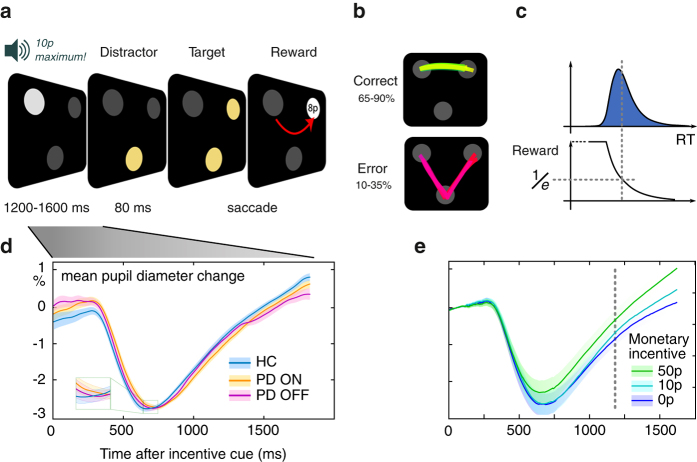
Measurement of autonomic response to reward cues. (**a**) In our task, participants were required to fixate an illuminated disc, and heard a recorded voice indicating how much reward could be won by making a speeded eye movement. Three reward levels were used: 0p; 10p; and 50p, randomized across 216 trials. After a 1,200–1,600 ms delay, a saccade had to be made to the second of two other discs that illuminated, one slightly later than the other. (**b**) Correct saccades were those that went directly to the target, whereas on error trials an initial saccade was made to the distractor. Percentages indicate the range of proportion of correct and error trials over all participants. (**c**) Reward was numerically displayed at the target, based on speed, and scaled up by the amount on offer on that trial. The value fell off exponentially with increasing response time (measured from distractor onset until gaze arrived at the target), with adaptive time constants that maintained a constant average rate of reward. (**d**) The pupil diameter was recorded at 1,000 Hz from the time of the cue for 1,800 ms. The pretrial baseline was subtracted, and average traces are shown over all trials for each group of participants. The shaded area indicates the standard error within subjects. No difference between groups was seen for the overall pupillary traces, as visible in the zoomed inset. (**e**) For the healthy controls, the pupil trace was averaged over trials of each reward level. Note that visually, there was no difference between reward levels. The three traces indicate that the pupil was more dilated after hearing a “50p” incentive, compared with “0p” or “10p” incentives (1p≈2 US cents).

Here we investigated whether pupillary reward responses in PD might reflect altered reward processing. In this monetary incentive task participants heard a reward precue, then had to make a speeded saccade, avoiding a distractor to obtain that reward (Figure 1a–c). Changes in pupil diameter in response to the incentive cue were examined in patients ON and OFF their normal medication.

## Results

Baseline pupil diameter was larger overall in patients than controls by 11% (*t*(36)=2.21, *P*=0.033), and was 4.9% larger when ON medication compared with OFF (*t*(15)=2.61, *P*=0.020). However, the size of the light response did not differ between patients and controls (*t*(36)=1.75, *P*>0.05), with no effect of medication (*P*>0.05; Figure 1d).

**Figure 2:**
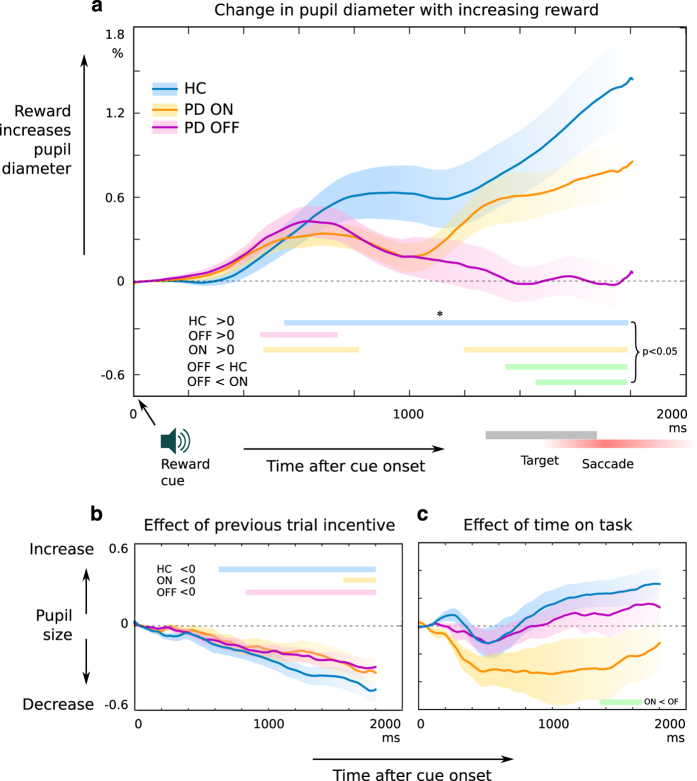
Effects on pupil size, given by linear regression at each time point. For each participant, the pupil traces were correlated with the incentive on the current trial, the incentive on the previous trial, and time on task. The coefficient of each of these factors was plotted as a function of time. (**a**) The influence of reward on pupil diameter is shown. Positive values indicate that with higher incentives, the pupil was larger; conversely negative values indicate that reward made the pupil smaller. Comparisons of these coefficients with zero, and with each other, are shown. Reward increased pupil size in all three groups, but controls were significantly more reward sensitive than PD patients when OFF medication (unpaired comparison). Also, PD patients were more reward sensitive when ON compared with when OFF (paired comparison). All statistics are calculated for *P*<0.05 controlling for family-wise error using permutation. (**b**) The previous trial’s incentive influenced the pupillary response to the subsequent incentive cue. Negative values signify that larger previous incentives result in a smaller pupil diameter on the current trial. There was a significant effect in all groups, and no significant differences between groups. (**c**) As the task progressed, pupillary dilatation effects might vary. To account for this, time on task (i.e., trial number) was included as a regressor. Positive values signify the pupillary response to the cue is more dilated later in the session, compared with earlier in the session. The value was not significantly different from zero in any group, although there was a late-in-trial (1,372–1,683 ms after cue) effect of medication, resulting in smaller pupils later in the session when ON compared with OFF. This might be attributable to increased fatiguability associated with D2 agonists. PD, Parkinson’s disease.

Pupil diameter at each time point after the auditory reward precue was analyzed using linear regression (Figure 1e), to calculate how incentive influenced pupil size. The slope of the reward term indicates how much the pupil size increased, relative to baseline, for an increase in reward level. A positive value indicates larger pupil diameter when more reward was on offer. The effect of reward is shown as a function of time (Figure 2a).

In controls, the effect of reward was positive: pupil size was larger after the high- compared with low-reward cues. This became significant 550 ms after the reward cue, and remained until the saccade (permutation correction for family-wise error at *P*<0.05, Supplementary Methods). A similar analysis OFF medication showed significant reward effects beginning >430 ms, but which now decayed, becoming nonsignificant >733 ms. Comparing patients OFF with healthy volunteers showed a significant reward-by-group interaction, with patients being less reward sensitive than controls after 1,334 ms. Patients’ pupils thus showed a dilatation with rewards, albeit with a shorter-lived response.

When tested ON, significant reward effect was observed after 439 ms, which remained significant until the target appeared. Comparison with controls showed no significant reward-by-group interaction, suggesting that ON medication, pupillary reward sensitivity was restored to normal levels. To demonstrate the effect of medication directly, pupillary reward effects were compared ON versus OFF medication, 1,200 ms post cue. A reward-by-drug interaction (F(1,15)=5.34, *P*=0.035) demonstrated that medication increased patients’ pupillary sensitivity to reward.

Delayed effects of reward were not affected, however. In both patients and controls, the previous trial’s incentive had negative effects on pupil size, with high previous reward leading to smaller pupils (Figure 2b), with no group differences. This indicates that longer term influences of reward were normal in PD.

Behavioral performance showed comparable distraction (error) rates ON and OFF (mean 22±3% vs. 19±2%; *t*(15)=0.13, *P*>0.05) and reaction times (366±26 ms vs. 376±30 ms, *t*(15)=0.59, *P*>0.05), suggesting pupillary effects of medication were not driven by changes in the need for cognitive control. In this study we found no evidence for saccade-related (Supplementary Figure S1) or distractibility-related pupillary changes (Supplementary Figure S2), although this experiment was not designed to detect these specific effects.

## Discussion

Previous research suggests altered reward processing in PD. This study is the first to quantify direct incentive effects via nonmotor measures. Reward sensitivity as measured by pupillometry was blunted OFF medication. This was restored to normal levels when ON, indicating that abnormal evaluation of rewards is coupled to dopaminergic function in PD.

These findings suggest a crucial role for dopamine in linking reward to motivational preparatory states. They support growing evidence in animals that dopamine has a dual role in evaluating reward and motivating effortful action.^12^ In PD, dopaminergic state also modulates motivational disturbances such as apathy and impulsivity, presumably by influencing incentive processing.^5,8^ Pupillometric measures might therefore provide a window into motivational abnormalities and their response to dopamine.

The drug- and disease-related effects observed here are unlikely to be caused by direct pupillomotor effects. Dopamine agonists produce inconsistent effects at the pupil, and the diminished and slow light reflex observed in PD^13^ is likely to be cholinergic and independent of dopaminergic state.^14^ Sympathetic output to the pupil appears to be relatively spared, as evidenced by normal responses to emotional faces in PD.^15^

Abnormal pupillary reward sensitivity in patients may arise through dysfunction of a brainstem arousal mechanism, in which dopamine and noradrenaline interact, linking reward to motivation. In addition to explaining how low-dopamine states alter reward-related learning and behavior, pupillary reward responses could provide a potential means to measure abnormal reward processing in the motivational syndromes of impulsivity and apathy in PD.

## Materials and methods

### Participants

Sixteen patients with mild-to-moderate idiopathic PD, mean age±s.d. (65.3±9 years), Unified Parkinson's Disease Rating Scale (UPDRS) 23.1±10.1, were tested (Supplementary Table S1). Patients were nondemented and nondepressed. Thirteen were on levodopa, and eight on a dopamine agonist (Supplementary Table S2). Patients were tested “ON” and “OFF” their normal medication, in two sessions in randomized order, and compared with 22 age-matched controls.

### Task

Participants performed an incentivized distractor-avoidance task (Figure 1a). Upon fixating an illuminated disc, they heard an auditory incentive cue that indicated how much money they could win on the upcoming saccade. After 1,200–1,600 ms, two other discs were illuminated, with 80-ms interval between the two onsets. Participants were required to make a saccade to the location that was illuminated second (the target), and avoid the first onset (distractor; Figure 1b). A numerical reward was displayed when gaze arrived at the target, and was proportional to both the initial incentive and speed (Figure 1c; Supplementary Methods). There were 216 trials, with 3 incentive levels in randomized order.
