# Supplementary material for: Reduced pupillary reward sensitivity in Parkinson’s disease
Source: NPJ Parkinsons Dis. 2015 Dec 17;1:15026–. doi: 10.1038/npjparkd.2015.26 (PMC5367517; doi:10.1038/npjparkd.2015.26)
Supplement: Supplementary Information [file npjparkd201526-s1.doc]

# Supplemental Methods

**Apparatus**

Stimuli were presented using Matlab and PsychToolbox on a PC and 17” CRT monitor at 1280x1024x100Hz. Participants were positioned on a chin-rest 60 cm from the monitor, in a darkened testing room. Eye movements were recorded at 1 kHz using a tower-mounted Eyelink 1000 infra-red eye tracker. The screen background was black with grey foreground. Screen locations were always indicated by dim grey discs, each 4° diameter, arranged in an equilateral triangle 11.4 degrees apart. Auditory cues were one of three voice recordings of the words “10p maximum” and the like, each 800 ms long and matched for amplitude, played over loudspeakers. The luminance of the fixation cue was 15.4 cd m^-2^. A non-ageing foreperiod of 1200-1600 ms separated the auditory cue and the distractor onset. The luminance of the distractor and target were 11.6 cd m^-2^.

**Participants**

Sixteen patients with mild-to-moderate idiopathic PD, mean age 65.3 s.d. 9, mean UPDRS 23.1, s.d. 10.1, who fulfilled the criteria for the Queen Square Brain Bank for PD were tested. The patients were non-demented (Montreal Cognitive Assessment > 25 or mini-mental state examination > 25). Thirteen were taking levodopa, and 8 were taking a dopamine agonist. Dopamine agonists comprised ropinirole (5 patients), pramipexole (2) and rotigotine (1). One patient was taking the antimuscarinic benzhexol, and one was taking the tricyclic antidepressant amitriptyline (**Table S1**). No patients were on antipsychotics or selective noradrenaline reuptake inhibitors. The Hospital Anxiety and Depression scale was used to screen for depression. Schwab and England ADL scores were 80% or above in 14 out of 16 patients, and was 60% and 50% in the other two patients. Patients attended on two days at least a week apart for ‘ON’ and ‘OFF’ sessions, in randomised order. On one session, they had omitted all dopaminergic medication for 12 hours prior to testing, and for the other session, they had taken their normal medication. Patients were compared with 22 age-matched controls recruited from an advert. All participants gave informed consent, and all research was conducted in accordance with UCL Research Ethics Committee guidance. The sample size was chosen based on prior estimates of pupillary reward sensitivity in healthy volunteers, giving an effect size of 1.05; a power of 0.8 14 participants.

**Reward schedule**

Rewards were calculated as an exponential fall-off as a function of the time for gaze to reach the target, from the distractor onset. The reward
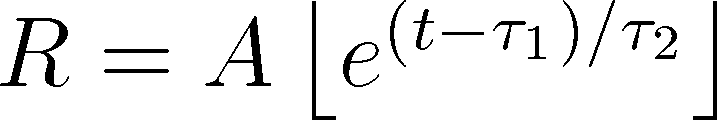
 depended on *A*, the total available reward available on that trial, and time constants
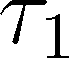
 and
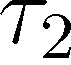
. Time constants were calculated adaptively from percentiles of the previous 20 trials, to keep 30% of trials slower than
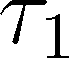
, and 10% of trials faster than
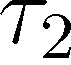
. This maintained a stable distribution of rewards over the duration of the task and across participants.

**Pupil analysis**

The pupil trace after the reward cue was preprocessed to remove blinks. If the blink was shorter than 500 ms, it was interpolated with a linear segment, and if the gap was longer, the trial was discarded. The baseline size for the 200 ms prior to cue onset was subtracted, and the proportional change in size relative to the baseline was calculated. The traces were smoothed using a 200 ms boxcar function. For each participant, we calculated the effect of the current trial’s incentive and the previous trial’s incentive, on pupil size. This was done using a general linear model (i.e. multiple regression) with incentive, previous incentive, and time on task as regressors. The latter regressor “de-trends” the effects to compensate for longer-term pupillary changes over the course of the experiment.

It is possible that using proportional changes relative to baseline might underestimate pupil dilatation if the resting pupil is near maximal size, due to ceiling effects. We therefore repeated the above analyses using the absolute change in pupil size. This demonstrated the same qualitative results as in **Fig. 2**, yielding significant differences between patients OFF and controls, and between ON and OFF states. Pupil baseline size and light reflex amplitude of the groups were compared using t-tests and were normally distributed according to Kolmogorov-Smirnov tests (all p>0.05).

**Statistics**

The above analysis resulted in coefficients of incentive, previous incentive and time on task as a function of time after the cue. To compare these pupil traces, we used permutation testing to correct for multiple comparisons. The traces of the two groups of interest were randomly permuted, and the *t*-statistic for each permutation was computed along the trace. The maximum value of this statistic along the whole trace was used, over 5000 permutations, to build a null distribution. A p-value could then be obtained by comparing the unpermuted t-statistics with this reference distribution, thus controlling the family-wise error rate.

**Saccade analysis**

Saccades were parsed online using criteria of acceleration >8000 deg/s^2^, velocity >30 deg/s, and amplitude >5 degrees. The first saccade that met these criteria after the onset of the distractor was used. If this saccade’s initial direction was within 30 deg of the target direction, the trial was classified as correct, whereas if it was within 30 deg of the direction of the distractor, the trial was deemed to be a distraction error. Other trials which included breaks of fixation and blinks, were rejected (7% in controls, 12% in patients).

# Supplemental Results

***1. Impulsivity***

Patients were specifically asked about symptoms of impulse control disorders, and none were reported. To quantify impulsivity more precisely, 15 of the 16 patients and 21 of 22 controls completed the UPPS Impulsive Behaviour Scale questionnaire^1^. There was no difference in impulsivity between the groups (mean score 28.3±12.1 s.d. in controls, 32.6±15.5 in patients, Wilcoxon rank sum p>0.05). To examine whether impulsivity related to reward responses, the pupil diameter reward sensitivity was calculated as the linear effect of reward on pupil size at 1400 ms after the cue. There was no correlation between UPPS score and pupillary reward sensitivity (controls: Spearman r=-0.15, PD ON r=0.03, PD OFF r=-0.01).

***2. Saccade-related pupillary changes***

To quantify any pupillary effects that were time-locked to the saccade, rather than to the cue, we first subtracted the cue-related trace. For each participant and foreperiod, the mean cue-related trajectory (calculated as in Fig. 1D) was subtracted from every trial to give residuals. This effectively removes any effects time-locked to the cue. The residual traces were then temporally re-aligned at the instant of the saccade (which differed on every trial). The re-aligned traces were averaged (Fig.S1) and demonstrate no significant pre-saccadic pupil change (traces not significantly different from zero, p>0.05). The re-aligned traces were also regressed against reward, to enquire whether the incentive effects observed in Fig.2 could be saccade-related. The absence of effects suggests (p>0.05) that the reward-related pupil changes are more likely to be cue-related than saccade related.

***3. Distraction***

To examine whether the pupillary response to the cue predicted whether the upcoming saccade would go to the distractor, rather than the target, we compared the average pupil dilatation on distracted trials, compared to correct trials (average traces shown in **Fig. S2A,** within-subject differences shown in **S2B**). There were no significant differences between the traces for controls, PD ON or PD OFF (all p>0.05 by permutation test).

***4. Secondary dilatation***

The pupil reactivity to light could be different across groups. Such reactivity differences could potentially confound our measures of reward sensitivity in Fig.2. To rule out this possibility, we tested whether individual differences in secondary dilatation modulated our measure of reward sensitivity.

For each trial, the point of maximum constriction, and point of maximum dilatation relative to baseline was found (i.e. the total height of the “upstroke” in **Fig.1D**). An individual’s secondary dilatation was calculated as the mean difference of maximum minus minimum across trials. Their reward sensitivity was calculated as the change in pupil diameter with reward level, at 1400 ms post-cue (calculated as in **Fig.2A**). Across all individuals, there was no correlation between the size of the secondary dilatation and reward sensitivity of pupil dilatation (r=0.14 p>0.05; **Fig.S3**). This demonstrates that the overall size of the secondary dilatation did not interact with the reward-related component of dilatation.

1. Whiteside, S. P. & Lynam, D. R. The Five Factor Model and impulsivity: using a structural model of personality to understand impulsivity. Personal Individ. Differ. 30: 669–689 (2001).

# Supplemental Tables and Figures

**Supplemental Table S1: Demographics of patients and age-matched controls**

| **PD Patients (n=19)** | mean | sd |
| --- | --- | --- |
| Age | 65.3 | 9.0 |
| UPDRS | 23.1 | 10.1 |
| Hoehn and Yahr Stage | 1.8 | 0.86 |
| Schwab and England daily living scale | 86% | 14% |
| Levodopa equivalent dose | 507 | 240 |
| HADS Depression score | 3.6 | 3.0 |
| Sex | 9 M | 10 F |
| **Matched Controls (n=22)** | | |
| Age | 62.5 | 8.9 |
| Sex | 10 M | 12 F |

**Supplemental Table S2: Individual patient medication**

|  | Levodopa | D2 agonist | Other PD treatment | Non-PD drugs |
| --- | --- | --- | --- | --- |
| 1 | Levodopa |  |  |  |
| 2 | Levodopa |  |  | Tamsulosin (antialpha) |
| 3 | Levodopa |  |  |  |
| 4 | Levodopa |  |  | Tolterodine (antimuscarinic) |
| 5 | Levodopa |  | Trihexyphenidyl (antimuscarinic) |  |
| 6 | Levodopa |  | Rasagiline |  |
| 7 | Levodopa |  | Rasagiline |  |
| 8 | Levodopa |  | Rasagiline | Insulin, Bendrofluazide |
| 8 | Levodopa | Pramipexole |  | Amitriptyline, Propranolol |
| 10 | Levodopa | Ropinirole |  | Propranolol |
| 11 | Levodopa | Ropinirole |  | Propranolol |
| 12 | Levodopa | Ropinirole |  |  |
| 13 | Levodopa | Ropinirole |  |  |
| 14 |  | Rotigotine | Rasagiline |  |
| 15 |  | Pramipexole |  | Prednisolone |
| 16 |  | Ropinirole | Rasagiline |  |


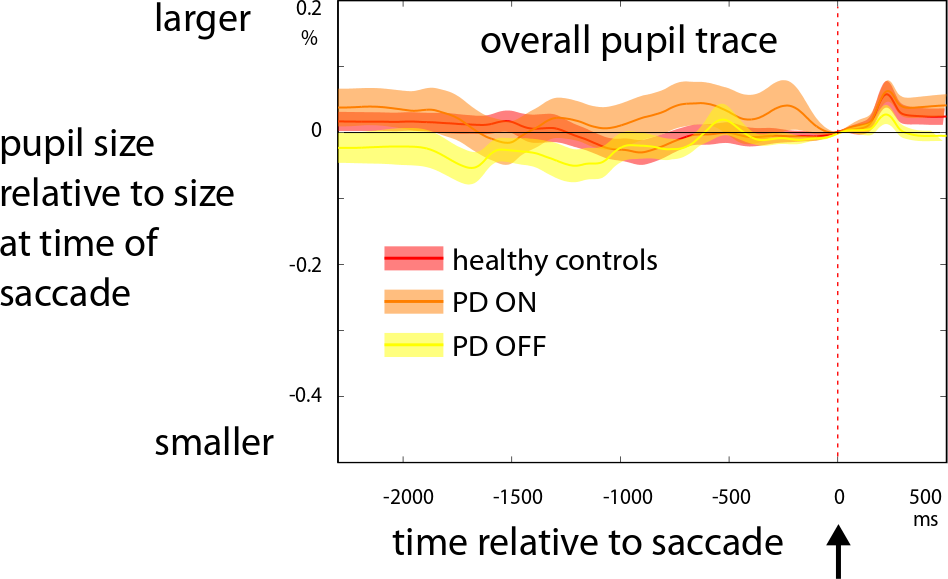


**Supplemental Figure S1: Pre-saccadic pupil diameter change**

The pupil traces were first aligned to the time of the cue, as in the main text. The cue-related response was then subtracted out, and the residual traces for each trial were re-aligned at the time of saccade. There was no significant presaccadic change in pupil diameter, that was not attributable to the cue. Shaded region is the standard error across subject mean traces.


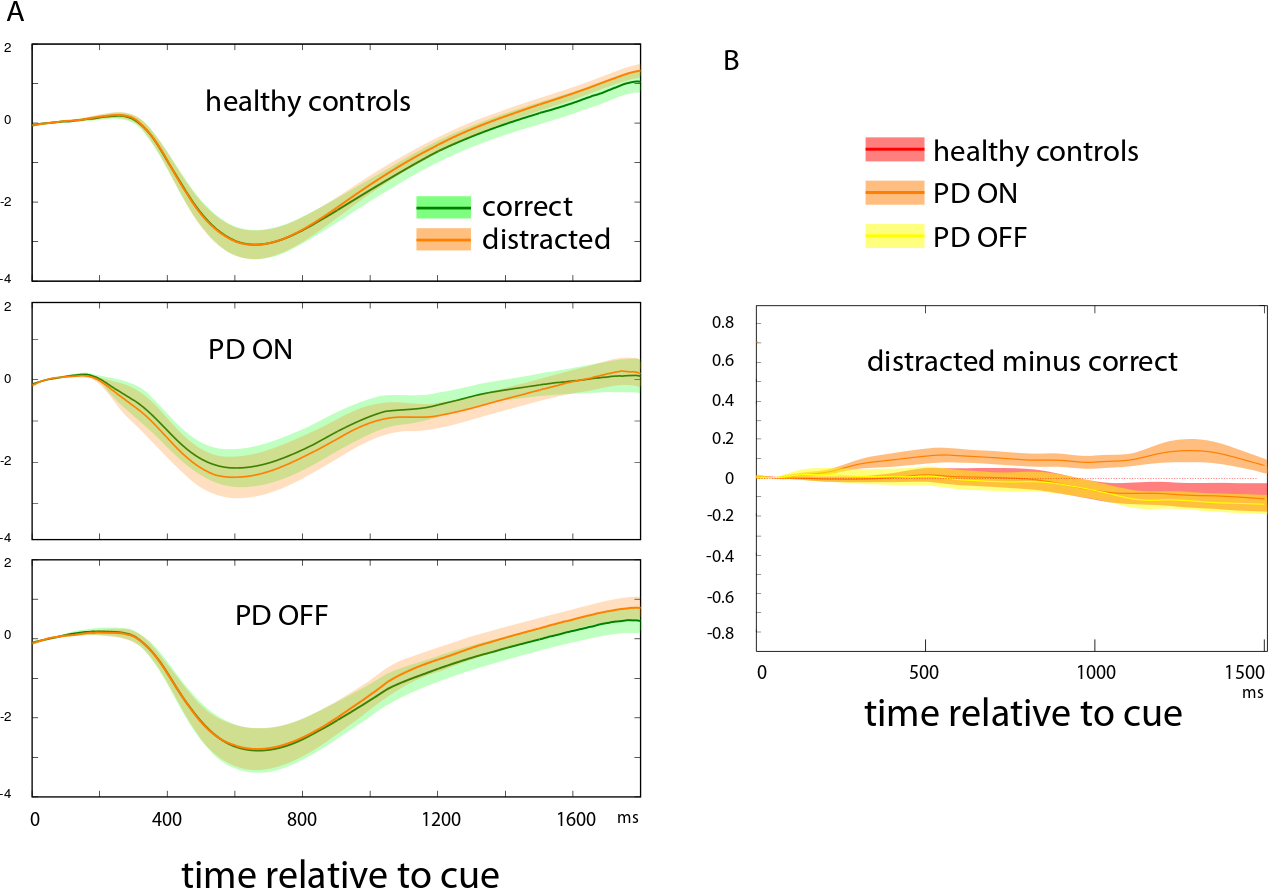


**Supplemental Figure S2: Effect of subsequent distraction on pupil traces**

A) Pupillary responses to the cue broken down according to whether the subsequent saccade went to the target or distractor. B) Difference traces between pre-distracted and pre-correct pupil responses. All groups showed no significant difference in pupillary response between distracted trials and correct trials (p>0.05 by permutation test)


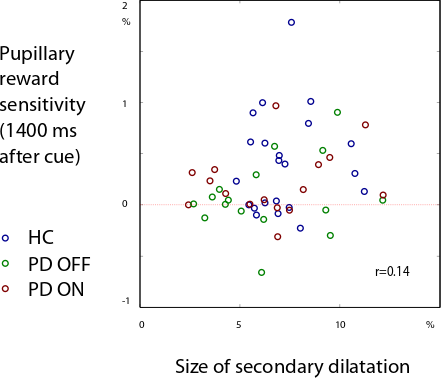


**Supplemental Figure S3: Reward sensitivity unrelated to size of secondary dilatation**

To ensure that reward sensitivity changes ON and OFF medication were not driven by changes in overall pupil reactivity, the overall size of the secondary pupil dilatation (total height of upward segment of trace in **Fig.1D**) was calculated for each participant session. There was no correlation between reward-related dilatation and overall amplitude of pupillary dilatation. Additionally no relationship was found in any group, indicating that the reward measure did not interact with the size of the light reflex.
